# Rehabilitation of an Extremely Resorbed Edentulous Mandible by Short and Narrow Dental Implants

**DOI:** 10.1155/2018/7597851

**Published:** 2018-12-20

**Authors:** Ines Kovacic, Sanja Persic, Josip Kranjcic, Nikolina Lesic, Asja Celebic

**Affiliations:** ^1^Department of Prosthodontics, School of Dental Medicine, University of Zagreb, Zagreb, Croatia; ^2^Prosthodontics at School of Dental Medicine, University of Zagreb, Dental Private Office, Zagreb, Croatia; ^3^Department of Prosthodontics, School of Dental Medicine & Clinical Hospital Centre Zagreb, University of Zagreb, Zagreb, Croatia

## Abstract

Serious consequences of long-term complete denture wearing may be extreme residual ridge atrophy and a reduced area of keratinized oral mucosa of a denture-bearing area. This paper presents five clinical cases of extreme mandibular ridge atrophy, rehabilitated by means of mandibular overdentures retained by short mini dental implants. The patients had a reduced mandibular bone volume in the interforaminal region, bone height less than 10 mm, and buccolingual bone width less than 4 mm. In order to avoid bone augmentation, patients received four short mini dental implants (MDIs) (6 or 8 mm long; 2.0 or 2.5 mm wide) for the support of mandibular overdentures, which is a new rehabilitation option. After insertion, the MDIs were early loaded with new mandibular overdentures reinforced with the CoCr framework. The patients have been wearing their overdentures for 2 years. One MDI broke during insertion and a new one was added. One patient lost one MDI but successfully continued to wear the overdenture retained by the remaining three MDIs. Mean marginal bone loss (MBL) was 0.20 ± 0.19 mm. Patients significantly improved their OHRQoL and chewing function by reducing the summary scores of the OHIP-14 and the chewing function questionnaires. The improvements remained unchanged throughout the observation period.

## 1. Introduction

Complete edentulism can be described as an irreversible condition that decreases oral health-related quality of life with consequences on chewing efficiency, nutrition, and general health. Although, the prevalence of complete edentulism has been decreasing in developed countries, it still remains a significant dental issue within the older population (depending on the country, 15% - 54.7% people older than 65 are completely edentulous) [1].

Years, or sometimes decades, of complete denture (CD) wearing may have adverse effects on the alveolar ridge bone, as well as on the keratinized attached mucosa of a denture-bearing area. Denture wearing, sometimes joined with poor bone quality, osteoporosis, or unstable mandibular denture, may result in extreme alveolar ridge atrophy [2–8]. Mandibular ridge atrophy can sometimes be so advanced that the mandibular height in the interforaminal region may be less than 10 mm (class D or E according to Lekholm and Zarb [9]), accompanied with a reduced buccolingual width and exposed inferior alveolar nerve under oral mucosa. In such cases, it is almost impossible to make a stable and well-functioning mandibular CD. Such a condition of extreme bone atrophy leads to chewing difficulties, pain, and sore spots within the denture-bearing area, resulting also in poor oral health-related quality of life (OHRQoL). Patients who have such difficulties are not satisfied with their dentures [2, 3, 10, 11].

Until a decade ago, the first treatment choice for edentulous patients was to make the maxillary and the mandibular complete denture. However, in 2002 the board of prosthodontists advised that mandibular overdenture supported by two standard size implants (SSI) should be the first treatment choice for mandibular edentulism (McGill consensus, 2002) [12].

Recently, four mini dental implants (MDI) minimum 10 mm long, inserted in the interforaminal region, have also been recommended as an appropriate treatment method for support of mandibular overdenture in edentulous patients with slim ridges (ITI consensus 2014) [13].

In rare cases of extreme mandibular ridge atrophy, mostly seen in patients wearing their complete dentures for a long time, it is impossible to insert SSIs due to a reduced buccolingual residual ridge width. It is also not possible to insert 10 mm long slim implants (MDIs) due to the reduced alveolar ridge height.

Such patients could be treated with a bone augmentation, which significantly prolongs duration of the treatment, and may not be successful. The procedure is time consuming, and patients often have some comorbidities due to their advanced age, which exclude extensive surgical procedures. Sometimes patients do not accept extensive treatments due to their fear of pain and possible complications. In one survey of edentulous patients who refused dental implant treatment, 66.7% patients indicated that the fear of pain was the main reason, followed by fear of the surgical procedures (64.8%), fear of postoperative complications (61.5%), and finally cost of implants (52.2%) [14].

One recent study revealed that flapless placement of MDIs caused significantly less pain than the insertion of SSIs [15].

In this clinical study, we treated patients who had reduced buccolingual width and reduced mandibular height (<10 mm) by means of implants being slim and short at the same time (short MDIs).

## 2. Case Presentations

### 2.1. Patient Selection

A total of 5 patients, 72 to 82 years old (4 females, one male), nonsmokers, participated. Four patients had a controlled hypertension, and one patient had a controlled diabetes type II. Patients were wearing their existing CDs from 6 to 11 years and were completely edentulous from 20 to 35 years. They were willing to improve their chewing function and esthetic outcome. All old existing dentures had poor retention and stability, and all patients had a reduced vertical dimension of occlusion. Four out of five patients had a persistent angular cheilitis.

**Figure 1:**
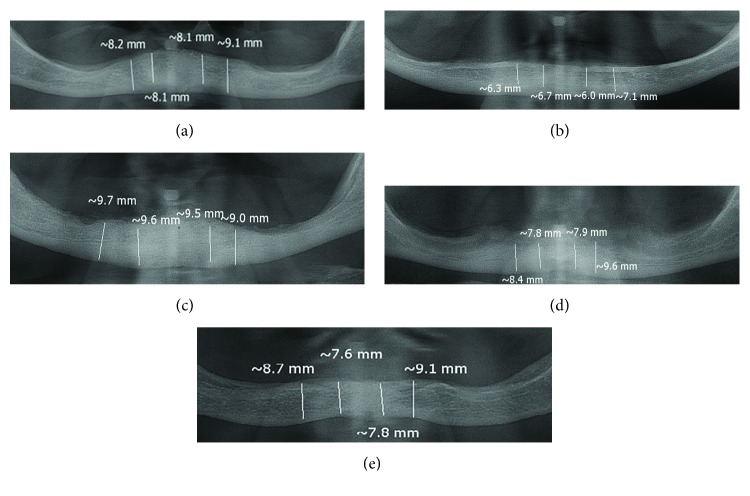
(a–e) Measurement of interforaminal mandibular height on panoramic radiographs of atrophied mandibles using the software SCANORA.

All patients were clinically examined. Their residual alveolar ridges in the mandible were excessively resorbed and atrophied (Figure 1); mental foramina were just below the mucosa overlying alveolar ridges.

**Figure 2:**
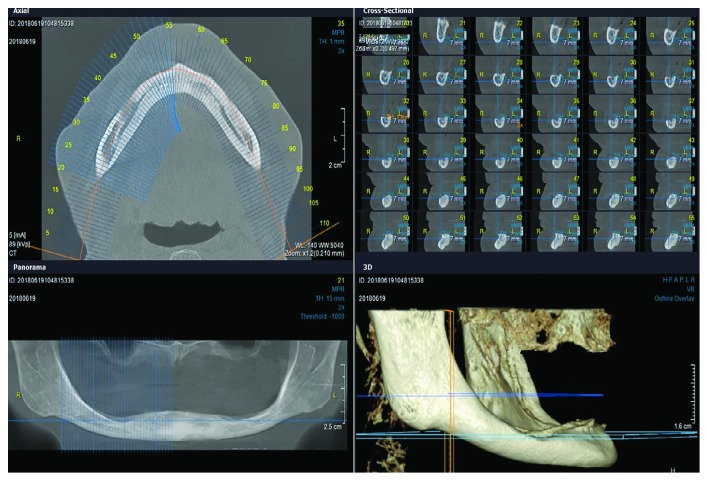
A CBCT scan of one patient with extensively resorbed mandibular alveolar ridge.

Panoramic radiographs and CBCT scans (Figure 2) revealed that the height of the mandible between the mental foramina was less than 10 mm with a reduced buccolingual width. The zone of attached mucosa overlying alveolar bone in the interforaminal region was equal to or less than 3.5 mm. Due to the patients' reduced buccolingual width, standard size implants or wide short implants could not be inserted without performing alveolar bone augmentation procedures. Due to the patients' old age and unwanted bone augmentation, we recommended the insertion of four short and slim MDIs (6 or 8 mm long) for the support of mandibular overdenture. The insertion of 10 mm MDIs having an intraosseous part of 8 mm has already been described [16]. Patients who had interforaminal height of the mandible > 9.0 mm received 8 mm long MDIs, and patients who had lesser height received 6 mm long MDIs. Patients whose residual ridge width was 3.1 mm or wider received 2.5 mm wide MDIs, while those with lesser ridge width received 2.0 mm wide MDIs.

The Ethics Committee of the Dental School of Medicine, University of Zagreb, Croatia, approved the protocol (no. 05-PA-26-6/2015), and all five patients signed a written informed consent form.

### 2.2. Surgical Procedure and Denture Manufacture

With the help of CBCTs and panoramic radiographs, insertion of four short MDIs in the interforaminal region was planned. The patients were prescribed with antibiotics prior to the surgical procedure. Each of the patients received 2 g of amoxicillin, one hour before implant surgery.

**Figure 3:**
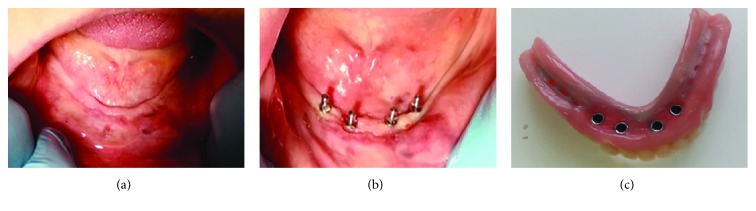
(a) Extensively resorbed denture-bearing area in the mandible; (b) four mini dental implants inserted; (c) mandibular overdenture reinforced with the CoCr framework with matrices and O-rings inserted.

**Figure 4:**
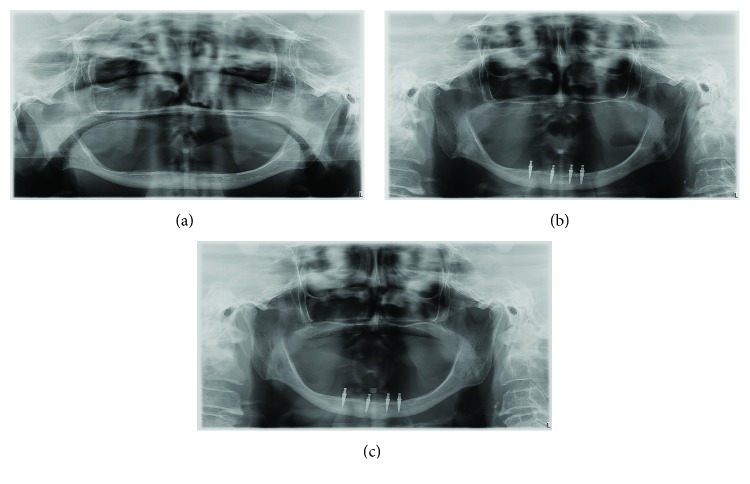
Panoramic radiographs (a) before treatment, (b) after one year, and (c) after 2 years of wearing the mandibular overdenture.

Each patient received four MDIs (Ti-6Al-4V, Dentium, Seoul, Korea; 2.0 or 2.5 mm wide, 6 or 8 mm long) in the interforaminal region without reflecting a flap (Figure 3(b)). All MDIs were inserted under local anesthesia (Ubistesin forte 3M ESPE, Germany) according to the manufacturer's instructions; calibrated burs (bur diameter: 1.3 for 2.0 mm wide MDI and 1.9 for 2.5 mm wide MDI) and a physiodispenser (W&H Implantmed, GmbH, Austria) with a saline solution for drill cooling were used. The depth of preparation for MDIs has been recommended to be one-third to two-thirds of the implant length [17]. All of the five patients had a very dense bone (D1 or D2, measurements obtained from CBCTs). Therefore, the preparation length for MDIs in our patients was equal to the whole mini-implants' intraosseous length. However, the bur diameter was smaller than the MDI diameter. Guide pins were used to evaluate parallelism of MDIs. Each MDI was inserted into the preparation hole and rotated clockwise exerting a downward pressure (self-tapping insertion technique), first using the thumb wrench and finally the torque wrench. All patients reached a final torque of >30 N/cm. During insertion one mini dental implant fractured due to an insertion torque > 45 N/cm and was left as a sleeping implant in the bone, while an additional MDI was inserted for proper denture retention (Figure 4(a)).

After the surgery an antiseptic mouth rinse (chlorhexidine gluconate 0.12% twice a day for 7 days) was prescribed and patients were provided with standard postsurgical instructions (cold ice packs during the first two postoperative days, nonsteroidal anti-inflammatory drugs, i.e., ibuprofen 400 mg, one hour after surgery and if necessary up to 7 days). Although the insertion torque for all implants exceeded 30 N/cm, the MDIs were early loaded (after 6-8 weeks). Immediately after MDI insertion, the holes were drilled in the old mandibular dentures, in order not to transfer any forces to the MDIs during the period of their osseointegration.

After 6 weeks the new mandibular overdentures were made in the dental laboratory and were delivered to patients. Individual impressions were obtained for each patient during denture manufacturing. When performing individual functional impression, with custom trays, thermoplastic compound was used for borders (ISO Functional, GC, Tokyo, Japan) and medium-viscosity silicone for the final impressions (Express^Tm^ Penta^Tm^, 3M ESPE, Seefeld, Germany). Transfer caps were used to enable the laboratory analogue placement and insertion of matrices with O-rings into the new mandibular denture. All new mandibular overdentures were reinforced with the CoCr framework to prevent denture fractures (Figure 3(c)). After denture processing and polishing, the new maxillary complete dentures and the new mandibular complete overdentures with O-ring matrices for denture retention were delivered to the patients. All denture adjustments were finished within the next two- to three-week period (excess denture material was removed at sore spot areas, minor occlusal adjustments were performed, etc.).

### 2.3. Primary Outcomes: Bone Loss and Technical Difficulties

The patients have now been wearing their dentures for two years. The control panoramic radiographs were made at the 1-year and 2-year observation stages (Figure 4).

The peri-implant bone was measured on panoramic radiographs (all panoramic radiographs were standardized and made on the same machine, CRANEX™ Novus e, Soredex, Tuusula, Finland), as it was not possible to place a CD sensor in the patients' sublingual area due to a shallow sublingual sulcus. During measurement, the magnification error was corrected using the following formula: corrected crestal bone level = (measured crestal bone level × actual implant length)/measured implant length (reported by Yoo et al. [18]). The MBL measurements were made under zoom-in using the SCANORA™ software 5.1. (Soredex, Tuusula, Finland).

### 2.4. Patient-Centered Outcomes

During the two-year period of the mandibular overdenture wearing, no matrix was changed, only two O-rings, one in each patient at the two-year follow-up stage.

**Figure 5:**
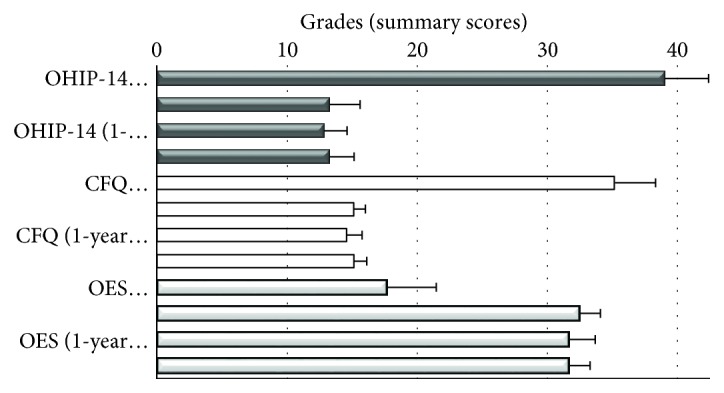
Mean summary scores of the OHIP-14, chewing function questionnaire (CFQ), and orofacial esthetic scale (OES) prior to the treatment, after receiving new dentures and finished adjustments, at the one-year follow-up examination and the 2-year follow-up clinical examination.

The patients also filled in the structured questionnaires describing their self-perceived oral health-related quality of life (OHRQoL) (the OHIP-14 questionnaire) [19], chewing function (chewing function questionnaire (CFQ)) [20], and orofacial esthetics (orofacial esthetic scale (OES)) [21]. The questionnaires were filled in four times: the first time prior to the treatment, the second time after receiving new dentures and adjustments finished, the third time at the one-year follow-up examination, and finally the fourth time at the 2-year follow-up clinical examination. The OHIP-14 questionnaire, as well as the CFQ were assessed by the Likert scale from 0-4; higher scores represented more pronounced difficulties. The OES questionnaire was assessed by the Likert scale ranging from 1 to 5 (higher scores represented better orofacial esthetics). Patients answered on each item regarding their experience during the last 7 days [22]. Pretreatment scores were compared with the after-treatment scores at the denture delivery stage (after adjustments) using the paired *t*-test. Repeated measurement tests (general linear model) were used to compare the postdelivery scores and the scores obtained at the 1-year and 2-year observation stages. The obtained results are presented in Figure 5.

## 3. Results

The mean marginal bone loss (MBL) in the remaining 19 MDIs was 0.20 ± 0.19 mm.

**Figure 6:**
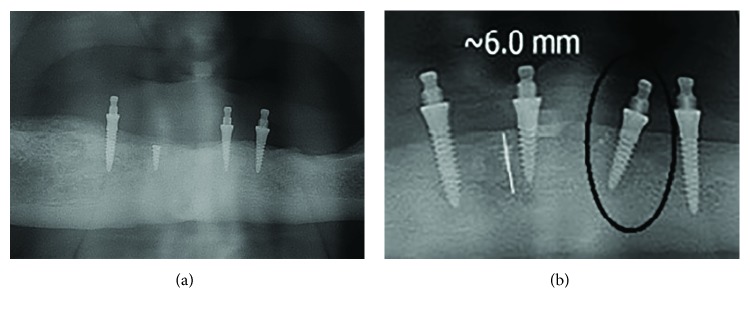
(a) One fractured MDI left as a sleeping implant; (b) one MDI that had to be removed (encircled).

One patient lost one MDI after one year and 6 months of denture wearing, but she has still been successfully wearing her mandibular overdenture retained by the three remaining short MDIs (Figure 6(b)).

The high values of the pretreatment baseline OHIP-14 summary scores decreased drastically after receiving and adjusting new mandibular overdentures retained by four short and slim implants (*t* = 19.56; *p* < 0.01). The summary score remained stable and almost unchanged over the two-year period (*p* = 0.59). The CFQ scores showed a similar trend. The high summary score registered at the baseline represented many chewing difficulties. Significant decrease of the summary score was observed after receiving new dentures (*t* = 17.45; *p* < 0.01). The summary score remained stable over the one-year and two-year periods of denture wearing (*p* = 0.908).

Patients' orofacial aesthetics received a significantly higher (better) summary score after treatment (*t* = −9.9; *p* = 0.01), as the vertical dimension of occlusion had been increased by new dentures. The OES score remained also stable and unchanged at the one- and two-year observation stages (*p* = 0.124).

## 4. Discussion

The five patients with an extremely atrophied mandible did not want a painful, long-lasting, and complicated surgical procedure of bone augmentation. Therefore, a minimally invasive protocol was chosen due to their advanced age. By inserting four slim and short implants (short MDIs) for the retention of mandibular overdenture, the vertical and the horizontal bone augmentation was avoided. The short and slim one-piece implants were inserted without raising a flap. This approach was selected in order to minimize patients' morbidity and surgical time.

The ITI [13] has already approved insertion of four MDIs of at least 10 mm length as a standard procedure. Such treatment is beneficial for elderly people, especially the ones with poor general health [23, 24]. One previous trial described the utilization of 10 mm MDIs with an intraosseous bone length of 8 mm to be a successful treatment option [16]; however, insertion of 6 mm long MDIs has not been used yet. Although it has been recommended to drill the preparation hole from one- to two-thirds of a mini-implant length [17], we had to drill the full implant length. All of the five patients had very dense bone, so one MDI broke during insertion due to too high insertion torque (insertion torque was over 50 N/cm). This MDI was left as a sleeping implant and another one was inserted nearby, so each of the patients received four functional short MDIs for mandibular overdenture retention.

Clinical success of short MDIs for retention of mandibular overdenture would be of tremendous benefit, as most patients with extreme alveolar ridge atrophy do not use their complete dentures at all, not even with denture adhesives, and most of them do not want extensive surgical treatments [25, 26].

In some cases of extreme mandibular atrophy, wide and short implants are not a perfect option due to a reduced buccolingual bone width. Even in patients with wider buccolingual alveolar bone width, insertion of short and wide implants may be questionable, as the preparation of mandibular bone for wide implants would leave only a slim cortical plate around implants after the insertion. The cortical plate has a reduced blood supply in comparison to the cancellous bone, so osseointegration would be slower, or even questionable. During the osseointegration period, any blow to the mandible may be a risk of bone fracture when only a thin cortical plate is left. However, after osseointegration, peri-implantitis may be another risk for bone fracture in such cases. Our patients received 4 short and slim MDIs, and the mandibular bone was preserved as much as possible. Mini-implants were inserted by drilling through the oral mucosa, through the cortical plate of the residual alveolar ridge, through the cancellous bone (if it was present), and through the beginning of the cortical bone of the lower edge of the mandible.

The status of the attached keratinized mucosa of a denture-bearing area is also very important for providing denture stability. The patients had a narrow area/zone of keratinized mucosa. The width of the keratinized mucosa of the denture-bearing area was about 3.5 mm. However, the mucosa height was less than 2.5 mm and it was not flabby, so the assumption was that the denture would be a stabile bearing area.

Maximum bite force (MBF) is low in CD wearers, especially in those with an atrophied mandible and poor denture-bearing area. It has been proven that standard length MDIs (≥10 mm) improve CD retention and OHRQoL and increase maximum bite force (MBF) [27]. Inserting implants for denture retention increase MBF from 120 N to 250 N [27]. One study showed that MBF was correlated with bone atrophy; the greater the bone atrophy was, the lower was the MBF [28, 29]. Individuals with natural teeth have MBFs that can exceed 600 N [29]. Our assumption was that short and slim MDIs would withstand patients' biting forces that are probably very low due to the ridge atrophy and much lower than in dentate subjects. However, insertion of 4 short MDIs probably increased patients' MBF, as they reported significantly low difficulties when chewing foods of different consistencies after the therapy, and it was consistent throughout the 2 years.

Clinical and radiographic follow-ups of our patients rehabilitated with 4 short MDIs and mandibular overdenture showed excellent preservation of marginal bone structure, as well as of healthy soft tissues, after the 12 and 24 months of wearing new dentures (Figure 4). It is similar to some other studies with standard size implants [29]. Obviously, slim but short MDIs helped to preserve remaining alveolar bone volume and enabled mandibular denture stability and its well function. During the overdenture-wearing observation period, none of the MDIs was broken, only one MDI was lost. The four slim and short implants showed the 95% survival and success rate. The 2-year period of only 0.20 mm mean marginal bone loss in the crestal MDI region is in accordance with the outcomes reported for standard length (10 mm or longer) MDIs [30, 31]. The survival rate with 4 MDIs is also similar, which was reported in the dental literature to be from 89.0 to 99.4% for the period of 1 to 5 years of overdenture wearing [16, 32, 33].

Although the manufacturer recommended that O-rings should be changed after one-year, only two O-rings were changed at the 2-year observation stage in the presented cases. The patients significantly improved both OHRQoL and chewing function. The scores of the both questionnaires (OHIP-14 and CFQ) remained stable throughout the 2-year period of clinical observation, presenting high patient satisfaction. With the mandibular overdenture of adequate stability, it was also possible to increase the height of the lower third of the face, which led to better self-reported orofacial esthetics in all 5 patients, which also remained unchanged within the 2 years.

To the best of our knowledge, the case series of patients rehabilitated with dental implants being slim and short, for the retention of mandibular overdenture, has only once been presented in the dental literature for 8 mm MDI intraosseous length [16], while it has not been presented for the 6 mm short MDIs. The presented cases were successfully rehabilitated with 4 short MDIs and showed high clinical and radiographic success. However, extensive clinical follow-up studies of a greater number of similar patients would be necessary throughout a longer time period to confirm or reject utilization of slim and short implants for retention of mandibular overdenture in all other cases of extreme mandibular bone atrophy.
